# Protein structure similarity from principle component correlation analysis

**DOI:** 10.1186/1471-2105-7-40

**Published:** 2006-01-25

**Authors:** Xiaobo Zhou, James Chou, Stephen TC Wong

**Affiliations:** 1 Harvard Center for Neurodegeneration and Repair – Center for Bioinformatics, Harvard Medical School, 1249 Boylston Street, Boston, MA 02215, USA; 2 Functional and Molecular Imaging Center, Radiology Department, Brigham and Women's Hospital, One Brigham Circle, 1620 Tremont Street, Boston, MA 02121, USA; 3 Department of Biological Chemistry and Molecular Pharmacology, Harvard Medial School, 240 Longwood Avenue, Boston, MA 02115, USA

## Abstract

**Background:**

Owing to rapid expansion of protein structure databases in recent years, methods of structure comparison are becoming increasingly effective and important in revealing novel information on functional properties of proteins and their roles in the grand scheme of evolutionary biology. Currently, the structural similarity between two proteins is measured by the root-mean-square-deviation (RMSD) in their best-superimposed atomic coordinates. RMSD is the golden rule of measuring structural similarity when the structures are nearly identical; it, however, fails to detect the higher order topological similarities in proteins evolved into different shapes. We propose new algorithms for extracting geometrical invariants of proteins that can be effectively used to identify homologous protein structures or topologies in order to quantify both close and remote structural similarities.

**Results:**

We measure structural similarity between proteins by correlating the principle components of their secondary structure interaction matrix. In our approach, the Principle Component Correlation (PCC) analysis, a symmetric interaction matrix for a protein structure is constructed with relationship parameters between secondary elements that can take the form of distance, orientation, or other relevant structural invariants. When using a distance-based construction in the presence or absence of encoded N to C terminal sense, there are strong correlations between the principle components of interaction matrices of structurally or topologically similar proteins.

**Conclusion:**

The PCC method is extensively tested for protein structures that belong to the same topological class but are significantly different by RMSD measure. The PCC analysis can also differentiate proteins having similar shapes but different topological arrangements. Additionally, we demonstrate that when using two independently defined interaction matrices, comparison of their maximum eigenvalues can be highly effective in clustering structurally or topologically similar proteins. We believe that the PCC analysis of interaction matrix is highly flexible in adopting various structural parameters for protein structure comparison.

## Background

Conformational resemblance between proteins, whether remote or close, is often used to infer functional properties of proteins and to reveal distant evolutionary relationships between two proteins exhibiting no similarity in their amino acid sequences. Traditionally, high-resolution structure determination succeeds the biological and biochemical studies of proteins to further provide mechanistic details of the function of proteins. The biological function of these proteins have usually been suggested prior to their structural studies by *in vitro *binding assays, *in vivo *gene knock-out experiments, and sequence homology with proteins of known function. However, with the completion of the sequencing of the genomes of human and other organisms, major structural biology resources have been harnessed to solve structures of large numbers of proteins encoded by the genomes in a high throughput but less specific fashion, under the name 'structural genomics' [1]. Subsequently, large sets of protein structures are accumulated in the public domain databases for which we know little about their biological roles. This shortfall calls for the development of cost-effective computational methods to predict protein function based on three-dimensional structures, with the aim of providing preliminary information to guide biological experiments later.

In the post-genomic era, large amounts of new protein sequences are available for statistics-based recognition of their biological properties. It has been shown in many cases that with the help of elegant computational algorithms, amino acid sequence information alone can be used to successfully predict a protein's structural class [2-4], sub-cellular location [5,6], and even enzymatic activities [7-10]. These approaches, however, are often limited by sequence noise arose from natural mutations throughout the evolutionary path, in which proteins are structurally and functionally conserved, but divergent in amino acid sequences. It is a recurring theme in structural biology that proteins with completely different sequences can adopt very similar global fold. Hence, incorporating structural information into functional genomics would potentially upgrade predictions to the next level of accuracy. Owing to the rapid technical advances in X-ray crystallography and liquid-state NMR spectroscopy, protein structure determination becomes more routine than before. It is reasonable to predict that full-scale structure determination can be the first step towards characterizing the biological role and mechanism of a newly sequenced protein. In the 13,000-large protein structure database (PDB), there are only approximately 4,000 different folds represented in the PDB, with a fold/structure ratio of approximately 1/5 (in the protein data bank) [11]. Therefore, given a new protein structure determined experimentally, chances are high that its topological arrangement of secondary fragments already exists in PDB either as an individual protein, or as a domain within a larger protein.

Structure comparison is traditionally based on coordinate RMSD [12,13]. While the RMSD approach is effective in comparing two close topologic structures with similar chain length, it fails when proteins are of different shapes or lengths. One outstanding example is Calmodulin, a ubiquitous Ca^2+ ^binding protein that plays a key role in numerous cellular Ca^2+^-dependent signaling pathways [14]. The backbone RMSD between the Ca^2+^-bound and apo states of individual calmodulin domain (~64 residues) is as large as 4**Å**, despite the fact that they are the same molecules with the same topology. When using the Ca^2+^-bound structure as a starting model, a homology based NMR residual dipolar coupling (RDC) refinement scheme, which relies heavily on the model having the correct topology, is able to converge the model to an accurate apo structure using RDCs measured for the apo state [15]. There are numerous proteins with similar secondary element arrangements in the 3D space yet acquire different overall shapes. Clearly for these proteins, algorithms different from the RMSD must be used to reveal their topological similarities. Another well-known software called Matching Molecular Models Obtained from Theory (MAMMOTH) is a sequence-independent protein structural alignment method [16]. It compares an experimental protein structure using an arbitrary low-resolution protein tertiary model. The distance defined in MAMMOTH is quite different from our approach. There are also many other methods of protein structure comparison, such as [17-21]. Note that all of the aforementioned methods used sequence based comparison. In contrast, our method adopts secondary structure based comparison and focuses on extracting invariant topological features.

In our study, we measure structural similarity between proteins by correlating the principle components of their secondary structure interaction matrix. In this method, referred here as the principle component correlation (PCC) analysis, the symmetric matrix for an individual protein is constructed with relationship parameters between secondary elements that can take the form of distance, orientation, or other relevant structural invariants. It is first demonstrated that the maximum eigenvalues of these interaction matrices can be effectively used to group structurally or topologically homologous proteins. Then by taking into account both maximum eigenvalues and their corresponding eigenvectors, a more refined pair-wise structure comparison is performed, which is able to differentiate structures of similar shape but different topological backbone traces. It is also shown that the results of PCC analysis are highly comparable to those given by the scaled Gauss metric (SGM) calculations [22] for the data sets studied. We believe the PPC method is flexible in adopting various structural parameters for pair-wise structure comparison.

## Results

### Materials

A total of fifty-six protein structures, grouped into 6 different sets according to CATH [23,24] are used to test our algorithms. Proteins in structure set I belong to the "mainly alpha" class, including mostly apoptosis regulators in the BCL-x_L _super family as well as others with remote conformational resemblance; all have the "Orthogonal Bundle" architecture. The atomic coordinates were retrieved from PDB with accession codes 1A4F, 1A6G, 1COL (A), 1DDB (A), 1F16 (A), 1G5M (A), 1GJH (A), 1MAZ, 1MDT (A), and 2BID (A), where (A) means chain A. Set II is also "mainly alpha" and has the same architecture as Set I, including structures 1CK7 (A), 1CXW (A), 1E8B (A), 1E88 (A), 1J7M (A), 1KS0 (A), 1PDC, and 2FN2. However this set consists of DNA helicase domains that have vastly different topology from Set I. Set II is used here to test the ability of our method to separate proteins that are in the same class of secondary structure but have different topologies. Set III belongs to the "mainly beta" class and has the barrel architecture, consisting of acid protease structures 1A5T, 1BVS (A), 1CUK, 1DV (A), 1F4I (A), 1G4A (E), 1G41 (A), 1HJP, 1IM2 (A), and 1JR3 (E). Set IV consists of the "alpha/beta" class proteins with the roll architecture, including structures 1FM0 (D), 1D4B (A), 1C78 (A), 1LM8 (B), 1NDD (A), 1UBQ, 1IBQ (A), and 1IP9 (A). The structures in set IV all have the Ubiquitin-like topology. Set V consists of the "mainly alpha" with the Alpha/alpha barrel architecture, including 1C82 (A), 1CB8 (A), 1EGU (A), 1F1S (A), 1F9G (A), 1HM2 (A), 1HM3 (A), 1HMU (A), 1HMW (A), 1HV6 (A), 1I8Q (A), and 1QAZ (A). The structures in Set V all have the Glycosyltransferase topology. Set VI consists of the "mainly beta" with the ribbon architecture, including 1AIW, 1E6N (A), 1E6P (A), 1E6R (A), 1E6Z (A), 1E15 (A), 1ED7 (A), and 1GOI (A). The structures in Set VI have the Seminal Fluid Protein PDC-109 (domain B).

### Clustering of structurally similar proteins by SMEC method

**Figure 2:**
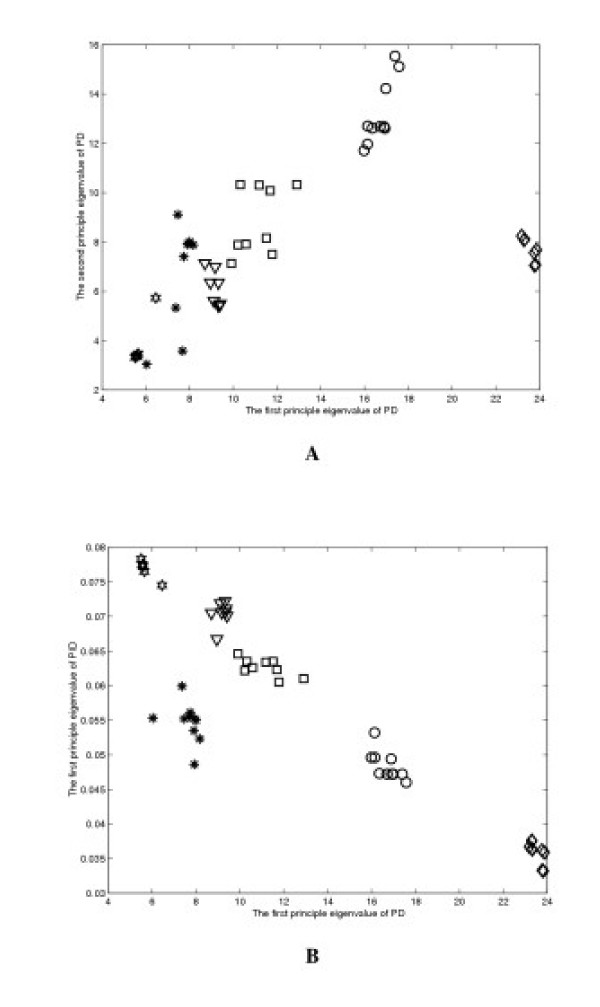
(a) The plot of scaled λ_2 _(the second largest eigenvalue) versus λ_1 _(the maximum eigenvalue), calculated using the PD matrix, for all proteins in the four data sets, and (b) the plot of λ_1 _of PID matrix versus that of PD matrices. The symbol representations are: ○ – structure set I; ▽ – set II;  – set III; □ – set IV; ☍ – set V; and  – set VI.

One of the goals of this study is to compare and identify structurally or topologically similar proteins. In other words, given a new experimentally determined protein structure, the proposed method is expected to rapidly place the structure into a group of structurally or topologically similar proteins in the database, thereby aiding in correlating topological similarity with functional similarity. To illustrate the application of the SMEC approach, we compute the scaled eigenvalues of PD and PID interaction matrices (Section Methods). Figure 2a shows the plot of scaled λ_2 _versus λ_1_, calculated using the PD matrix, for all proteins in the four data sets. Figure 2b shows the plot of λ_1 _of PID matrix versus that of PD matrices. The different symbols represent different structural groups. These plots were used to resolve clusters of structurally similar structures.

### Pair-Wise structural comparison by PCC method

In addition to correlating the maximum eigenvalues, the PCC method described in Section Methods, which compares both eigenvalues and eigenvectors, was tested for the four selected data sets. Using the pair-wise distance matrix defined in Section Methods, the difference metric *R *defined in Eq. 5 between all pairs of protein structures in the four data sets were calculated and shown in Tables 1-6. Additionally for the same data sets, writhing numbers computed using the SGM method were presented in the same corresponding tables. The *R *values between a few selected proteins from different groups were also shown to provide a negative control (Table 2).

**Table 2:** Pair-wise *R *values calculated using the PD matrix between representative structures from different structure sets.

	2BID	1C78	2FN2
1A5T	2.1121	6.8168	5.8935
1C78		4.6893	8.3020
1FN2			7.6954

## Discussion

**Figure 3:**
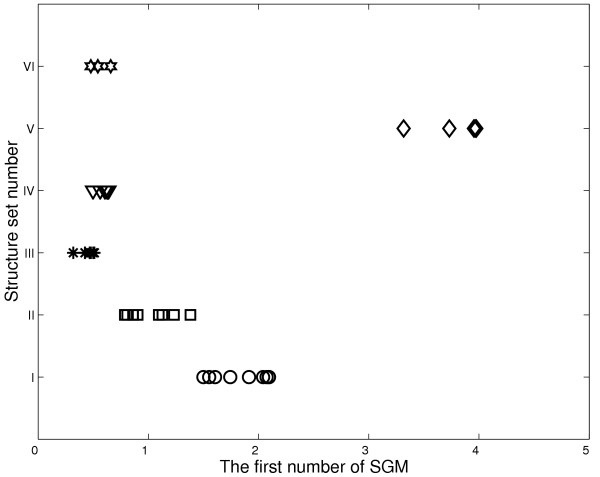
The first number of SGM of proteins in all four structural sets. The symbol representations are the same as in Figure 2.

The concept of principle component analysis (PCA) is widely used in mathematics and pattern recognition to simplify a data set. In mathematical terms, it is a transform that chooses a new coordinate system for the data set, such that the greatest variance by any projection of the data set comes to lie on the first axis (then called the first principle component), the second greatest variance on the second axis, and so on. Because of the large amount of information stored along the first axis, the maximum eigenvalue itself can be characteristic enough to represent structural features of a protein. Figure 2a plots eigenvalues λ_1 _versus λ_2 _derived from the PD matrices of all four sets of structures under study. Clearly λ_1 _values alone are distinct enough from each other for grouping most of the structures into their known conformation sets. The same plot also illustrates that the second largest eigenvalue λ_2 _is generally not powerful enough to accomplish the grouping. It is therefore expected that smaller components of interaction matrices are not effective for this purpose. Similarly, when using the first number computed with the SGM algorithm, the four structure sets can be resolved (see Fig. 3).

In addition to the PD matrix, PID matrix defined above was used to provide further separation between clusters of eigenvalues. This was demonstrated in Fig. 2b, in which the plot of λ_1 _of PID matrices versus that of PD matrices achieves a much better grouping of the four structural sets in the vertical dimension as compared to the plot in Fig. 2a. This further emphasizes the importance of the maximum eigenvalues and variations in the definition of the interaction matrix that provides independent structural information. It does not escape our notice that even better resolution can be achieved by correlating λ_1 _with three or more different types of interaction matrices in a multi-dimensional plot. The caveat, however, is that definitions of invariant relation constructing the matrices should not be redundant as there are a limited number of independent invariants in a protein structure. Nevertheless, the results here show that the PCA method using secondary interaction matrix is highly flexible in adopting various structural parameters as a means of structure comparison. We also investigate how much the first eigenvalue captures the eigenvalue spectrum in the BCL-x_L _family. We found that the first eigenvalue captures 45.78% of the sum of the 105 eigenvalues. That indicates that more eigenvalues could be helpful in protein structure classification in our future work.

**Figure 5:**
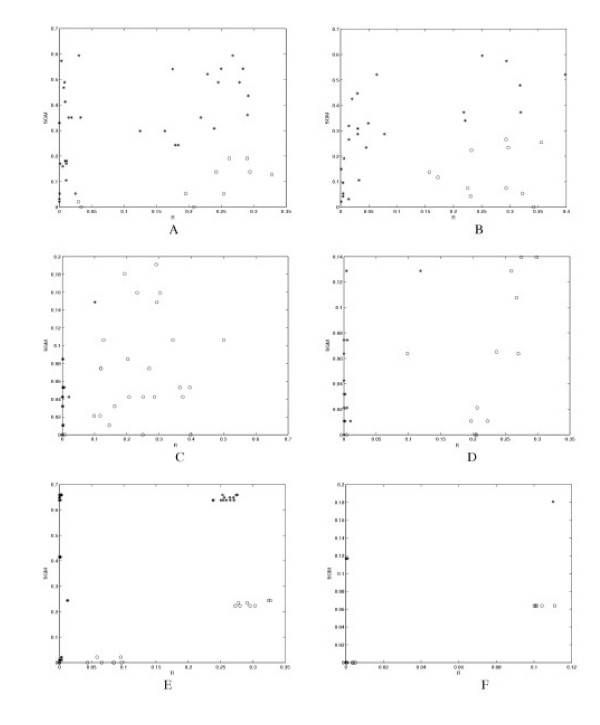
The plot of R score versus the SGM score: (a)-(f) are plotted for datasets from I to VI, respectively. The SGM score is defined as the absolute difference between the SGM values of two proteins. The symbol '*' denotes that the R score is smaller than SGM score, and the 'o' denotes the R score is bigger than SGM score.

A more elaborate method built on PCA is explored in this study to utilize the directional information contained in the eigenvector corresponding to λ_1_, named here as the PCC analysis as described in Section Methods. This method is particularly suited for the pair-wise structural comparison. Using the simple PD matrix definition (Section Methods), the pair-wise difference metrics, *R*, are all small (< 0.4) within each of the four known structural sets (Tables 1 and Figure 5(a)–(f)). The SGM score in Figure 5 is defined as the absolute difference between the SGM values of two proteins. The symbol 'o' denotes that the R score is smaller than SGM score, and the '*' denotes the R score is bigger than SGM score. Furthermore, as a negative control, *R *values between structures from different sets are much larger, typically greater than 2.0 (Figure 5(e)). Based on the *R *values in Table 1 and Figure 5(a)–(f) , we found empirically that by setting the cutoff *R *value to 0.4, the PCC method can faithfully place all structures in their designated groups.

**Table 1:** Pair-wise *R *values calculated using the PD matrix and the first number of SGM for proteins in structure set I.

		1F16	1G5M	1GJH	1MAZ	1DDB	1MDT	1COL	1A6G	1A4F
2BID	PCC	0.0249	0.0188	0.2185	0.2676	0.0000	0.0093	0.0337	0.2452	0.2835
	SGM	0.0530	0.3510	0.3510	0.5940	0.0210	0.1810	0.0031	0.4890	0.5420
1F16	PCC		0.1630	0.1248	0.1750	0.0000	0.3280	0.0005	0.2915	0.2780
	SGM		0.2980	0.2980	0.5410	0.0320	0.1280	0.0530	0.4360	0.4890
1G5M	PCC			0.2077	0.1836	0.0000	0.0013	0.0145	0.2943	0.2624
	SGM			0.0005	0.2430	0.3300	0.1700	0.3510	0.1380	0.1910
1GJH	PCC				0.1790	0.0000	0.0109	0.0327	0.2421	0.2899
	SGM				0.2430	0.3300	0.1700	0.3510	0.1380	0.1910
1MAZ	PCC					0.0031	0.0092	0.0303	0.0107	0.2537
	SGM					0.5730	0.4130	0.5940	0.1050	0.0520
1DDB	PCC						0.0054	0.0293	0.0068	0.2286
	SGM						0.1600	0.0210	0.4680	0.5210
1MDT	PCC							0.0112	0.2390	0.2904
	SGM							0.1810	0.3080	0.3610
1COL	PCC								0.0081	0.2496
	SGM								0.4890	0.5420
1A6G	PCC									0.1950
	SGM									0.0530

To provide a more in-depth view of the PCC method, the analysis of data set I is described here in detail. This set consists of mainly α helical structures having the "Orthogonal Bundle" architecture. Proteins 2BID, 1F16, 1G5M, 1GJH, 1MAZ, and 1DDB are apoptosis regulators of cell-death pathways associated with mitochondrion. Since mitochondria originated from prokaryotes, these proteins are believed to have evolved from the same ancient design. Although they differ substantially in amino acid sequence as well as in shape, the overall scaffold and topology are similar. As expected, the *R *values among them are all less than 0.4 (Table 1). Other proteins in this set, including bacterial toxins that are capable of forming membrane pores (1MDT and 1COL) and myoglobin (1A6G), have remote conformational resemblance with the BCL-x_L _proteins. The *R *values between these structures and the apoptosis regulators are also less than 0.3 and are comparable to those found within the BCL-x_L _family. It is interesting to note that although 1MDT and 1COL are not related to the BCL-x_L _proteins in terms of physiological roles, they do share a similarity with the BCL-x_L _members other than topology; that is, they all are able to form large pores when inserted into cellular membrane.

In summing the results of Table 1 and Figure 5(a)–(f), the *R *values within individual sets are on average very small, with a mean of 0.1102 and standard deviation of 0.1269. This is expected because the structures have been manually examined and pre-grouped into topologically similar sets. The comparison results from PCC analyses are generally comparable to that of SGM for the data sets under study (see Table 1 and Figure 5(a)–(f)). However, in a few isolated cases, the difference in the scaled writhing numbers within the same structure set can exceed the threshold of 0.4 that governs similarity (for example, protein pairs (1MAZ, 2BID), (1F16, 1DDB) in Table 1, and protein pairs (1C78, 1FM0), (1C78, 1NDD), and (1C78, 1IBQ) in Figure 5(b). This is because the PCC analysis using the PD matrix emphasizes more on spatial separation and orientation of secondary segments. It must be mentioned that the PD matrix alone is not expected to detect pure topological similarities. The results for structure sets with predominately β strands and mixed α/β proteins show similar *R *values (Figure 5(c) and 5(d)), indicating the generality of this method in protein structure comparison. We also tested these six data sets using MAMMOTH, it can also separate the six classes well.

**Figure 4:**
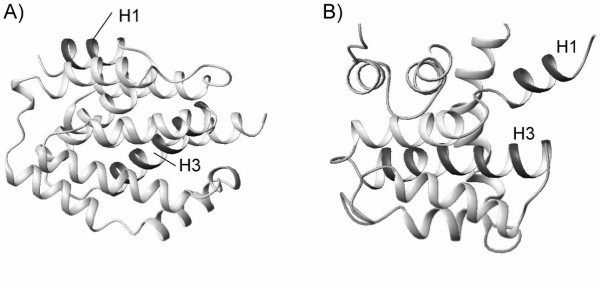
Ribbon representation of protein structures of (a) 1COL and (b) 1DDB. The two proteins have similar shape, but different topological arrangements in helices 1 and 3.

Another variation of the PD matrix definition is to take into account the N – C terminal sense, in attempt to further emphasize protein topological features. A good example is the comparison between structures 1COL and 1DDB in data set I. A visual examination of the two structures reveals that they share similar shape, but are considerably different in topological arrangement of helices 1 and 3. In protein 1COL, the first and third helices are anti-parallel, whereas they are parallel in 1DDB (see Figure 4). This is not identified by the PCC analysis using the PD matrix as *R *= 0.029. The great similarity in shape prevailed in the comparison. However, by applying the PDS matrix defined in Section Methods, the *R*-value considerably increases to 1.707, clearly highlighting the difference in backbone topological traces. Finally we also would like to pint out that the definition of *R *could be improved by introducing more eigenvalues.

## Conclusion

PCC analysis of secondary interaction matrix is a conceptually simple method that yields results highly comparable to the SGM method. Both are able to distinguish protein conformations based on the more subtle topological features. While the SGM method compares structures in a more topological sense, the outcome of PCC analysis is more dependent on the definition of the interaction matrix. With the PD matrix, the PCC analysis puts more weight on the detailed structure and shape, while it is also capable, to a certain extent, of distinguishing different topological traces. In certain cases of pair-wise comparison, such as that between 1COL and 1DDB, protein shapes can overwhelm their topological features in the analysis; yet the PCC analysis of the PDS matrix is able to completely differentiate between 1COL and 1DDB. Owing to the flexibility offered by the new method, a more effective definition of interaction matrix can be explored to provide a more efficient structure comparison. There exist many invariants in each protein. Some invariants are important for protein classification, but some are not. Hence, our future work will further explore feature selection, automated classification of PDB, modeling and statistical learning, as well as protein domain matching.

## Methods

### Principle component analysis of secondary interaction matrix

Assuming a protein having *n *secondary fragments denoted by **h**_1_, **h**_2_,..., **h**_*n*_, and the number of residues in each secondary structure denoted by *l*_1_, *l*_2_,..., *l*_*n*_, respectively, the total number of residues belonging to secondary structures is given by N=∑i=1nli
 MathType@MTEF@5@5@+=feaafiart1ev1aaatCvAUfKttLearuWrP9MDH5MBPbIqV92AaeXatLxBI9gBaebbnrfifHhDYfgasaacH8akY=wiFfYdH8Gipec8Eeeu0xXdbba9frFj0=OqFfea0dXdd9vqai=hGuQ8kuc9pgc9s8qqaq=dirpe0xb9q8qiLsFr0=vr0=vr0dc8meaabaqaciaacaGaaeqabaqabeGadaaakeaacqWGobGtcqGH9aqpdaaeWbqaaiabdYgaSnaaBaaaleaacqWGPbqAaeqaaaqaaiabdMgaPjabg2da9iabigdaXaqaaiabd6gaUbqdcqGHris5aaaa@38AC@. The invariant relation between a pair of secondary elements (**h**_*i*_, **h**_*j*_) is described by a block matrix **F**(**h**_*i*_, **h**_*j*_), in which the individual matrix elements represent a particular relation between residues of the two secondary structures. Since **h**_*i *_has *l*_i _residues (denoted by ci1
 MathType@MTEF@5@5@+=feaafiart1ev1aaatCvAUfKttLearuWrP9MDH5MBPbIqV92AaeXatLxBI9gBaebbnrfifHhDYfgasaacH8akY=wiFfYdH8Gipec8Eeeu0xXdbba9frFj0=OqFfea0dXdd9vqai=hGuQ8kuc9pgc9s8qqaq=dirpe0xb9q8qiLsFr0=vr0=vr0dc8meaabaqaciaacaGaaeqabaqabeGadaaakeaacqWGJbWydaqhaaWcbaGaemyAaKgabaGaeGymaedaaaaa@3073@, ci2
 MathType@MTEF@5@5@+=feaafiart1ev1aaatCvAUfKttLearuWrP9MDH5MBPbIqV92AaeXatLxBI9gBaebbnrfifHhDYfgasaacH8akY=wiFfYdH8Gipec8Eeeu0xXdbba9frFj0=OqFfea0dXdd9vqai=hGuQ8kuc9pgc9s8qqaq=dirpe0xb9q8qiLsFr0=vr0=vr0dc8meaabaqaciaacaGaaeqabaqabeGadaaakeaacqWGJbWydaqhaaWcbaGaemyAaKgabaGaeGOmaidaaaaa@3075@,..., cili
 MathType@MTEF@5@5@+=feaafiart1ev1aaatCvAUfKttLearuWrP9MDH5MBPbIqV92AaeXatLxBI9gBaebbnrfifHhDYfgasaacH8akY=wiFfYdH8Gipec8Eeeu0xXdbba9frFj0=OqFfea0dXdd9vqai=hGuQ8kuc9pgc9s8qqaq=dirpe0xb9q8qiLsFr0=vr0=vr0dc8meaabaqaciaacaGaaeqabaqabeGadaaakeaacqWGJbWydaqhaaWcbaGaemyAaKgabaGaemiBaW2aaSbaaWqaaiabdMgaPbqabaaaaaaa@326C@), and **h**_*j *_has *l*_j _residues (denoted by cj1
 MathType@MTEF@5@5@+=feaafiart1ev1aaatCvAUfKttLearuWrP9MDH5MBPbIqV92AaeXatLxBI9gBaebbnrfifHhDYfgasaacH8akY=wiFfYdH8Gipec8Eeeu0xXdbba9frFj0=OqFfea0dXdd9vqai=hGuQ8kuc9pgc9s8qqaq=dirpe0xb9q8qiLsFr0=vr0=vr0dc8meaabaqaciaacaGaaeqabaqabeGadaaakeaacqWGJbWydaqhaaWcbaGaemOAaOgabaGaeGymaedaaaaa@3075@, cj2
 MathType@MTEF@5@5@+=feaafiart1ev1aaatCvAUfKttLearuWrP9MDH5MBPbIqV92AaeXatLxBI9gBaebbnrfifHhDYfgasaacH8akY=wiFfYdH8Gipec8Eeeu0xXdbba9frFj0=OqFfea0dXdd9vqai=hGuQ8kuc9pgc9s8qqaq=dirpe0xb9q8qiLsFr0=vr0=vr0dc8meaabaqaciaacaGaaeqabaqabeGadaaakeaacqWGJbWydaqhaaWcbaGaemOAaOgabaGaeGOmaidaaaaa@3077@,..., cjlj
 MathType@MTEF@5@5@+=feaafiart1ev1aaatCvAUfKttLearuWrP9MDH5MBPbIqV92AaeXatLxBI9gBaebbnrfifHhDYfgasaacH8akY=wiFfYdH8Gipec8Eeeu0xXdbba9frFj0=OqFfea0dXdd9vqai=hGuQ8kuc9pgc9s8qqaq=dirpe0xb9q8qiLsFr0=vr0=vr0dc8meaabaqaciaacaGaaeqabaqabeGadaaakeaacqWGJbWydaqhaaWcbaGaemOAaOgabaGaemiBaW2aaSbaaWqaaiabdQgaQbqabaaaaaaa@3270@), the elements of the *l*_*i *_× *l*_*j *_**F **block matrix, *g*(ciu
 MathType@MTEF@5@5@+=feaafiart1ev1aaatCvAUfKttLearuWrP9MDH5MBPbIqV92AaeXatLxBI9gBaebbnrfifHhDYfgasaacH8akY=wiFfYdH8Gipec8Eeeu0xXdbba9frFj0=OqFfea0dXdd9vqai=hGuQ8kuc9pgc9s8qqaq=dirpe0xb9q8qiLsFr0=vr0=vr0dc8meaabaqaciaacaGaaeqabaqabeGadaaakeaacqWGJbWydaqhaaWcbaGaemyAaKgabaGaemyDauhaaaaa@30F6@, cjv
 MathType@MTEF@5@5@+=feaafiart1ev1aaatCvAUfKttLearuWrP9MDH5MBPbIqV92AaeXatLxBI9gBaebbnrfifHhDYfgasaacH8akY=wiFfYdH8Gipec8Eeeu0xXdbba9frFj0=OqFfea0dXdd9vqai=hGuQ8kuc9pgc9s8qqaq=dirpe0xb9q8qiLsFr0=vr0=vr0dc8meaabaqaciaacaGaaeqabaqabeGadaaakeaacqWGJbWydaqhaaWcbaGaemOAaOgabaGaemODayhaaaaa@30FA@), are defined as

g(ciu,cjv)={d(ciu,cjv)i≠j0i=j,     (1)
 MathType@MTEF@5@5@+=feaafiart1ev1aaatCvAUfKttLearuWrP9MDH5MBPbIqV92AaeXatLxBI9gBaebbnrfifHhDYfgasaacH8akY=wiFfYdH8Gipec8Eeeu0xXdbba9frFj0=OqFfea0dXdd9vqai=hGuQ8kuc9pgc9s8qqaq=dirpe0xb9q8qiLsFr0=vr0=vr0dc8meaabaqaciaacaGaaeqabaqabeGadaaakeaacqWGNbWzcqGGOaakcqWGJbWydaqhaaWcbaGaemyAaKgabaGaemyDauhaaOGaeiilaWIaem4yam2aa0baaSqaaiabdQgaQbqaaiabdAha2baakiabcMcaPiabg2da9maaceaabaqbaeqabiGaaaqaaiabdsgaKjabcIcaOiabdogaJnaaDaaaleaacqWGPbqAaeaacqWG1bqDaaGccqGGSaalcqWGJbWydaqhaaWcbaGaemOAaOgabaGaemODayhaaOGaeiykaKcabaGaemyAaKMaeyiyIKRaemOAaOgabaGaeGimaadabaGaemyAaKMaeyypa0JaemOAaOgaaaGaay5EaaGaeiilaWIaaCzcaiaaxMaadaqadaqaaiabigdaXaGaayjkaiaawMcaaaaa@55C8@

where 1 ≤ *u *≤ *l*_*i*_, 1 ≤ *v *≤ *l*_*j*_, and *d*(ciu
 MathType@MTEF@5@5@+=feaafiart1ev1aaatCvAUfKttLearuWrP9MDH5MBPbIqV92AaeXatLxBI9gBaebbnrfifHhDYfgasaacH8akY=wiFfYdH8Gipec8Eeeu0xXdbba9frFj0=OqFfea0dXdd9vqai=hGuQ8kuc9pgc9s8qqaq=dirpe0xb9q8qiLsFr0=vr0=vr0dc8meaabaqaciaacaGaaeqabaqabeGadaaakeaacqWGJbWydaqhaaWcbaGaemyAaKgabaGaemyDauhaaaaa@30F6@, cjv
 MathType@MTEF@5@5@+=feaafiart1ev1aaatCvAUfKttLearuWrP9MDH5MBPbIqV92AaeXatLxBI9gBaebbnrfifHhDYfgasaacH8akY=wiFfYdH8Gipec8Eeeu0xXdbba9frFj0=OqFfea0dXdd9vqai=hGuQ8kuc9pgc9s8qqaq=dirpe0xb9q8qiLsFr0=vr0=vr0dc8meaabaqaciaacaGaaeqabaqabeGadaaakeaacqWGJbWydaqhaaWcbaGaemOAaOgabaGaemODayhaaaaa@30FA@) is a real number representing an arbitrary invariant relation between residues of **h**_*i *_and **h**_*j*_. Note this approach allows the definition of *d*(ciu
 MathType@MTEF@5@5@+=feaafiart1ev1aaatCvAUfKttLearuWrP9MDH5MBPbIqV92AaeXatLxBI9gBaebbnrfifHhDYfgasaacH8akY=wiFfYdH8Gipec8Eeeu0xXdbba9frFj0=OqFfea0dXdd9vqai=hGuQ8kuc9pgc9s8qqaq=dirpe0xb9q8qiLsFr0=vr0=vr0dc8meaabaqaciaacaGaaeqabaqabeGadaaakeaacqWGJbWydaqhaaWcbaGaemyAaKgabaGaemyDauhaaaaa@30F6@, cjv
 MathType@MTEF@5@5@+=feaafiart1ev1aaatCvAUfKttLearuWrP9MDH5MBPbIqV92AaeXatLxBI9gBaebbnrfifHhDYfgasaacH8akY=wiFfYdH8Gipec8Eeeu0xXdbba9frFj0=OqFfea0dXdd9vqai=hGuQ8kuc9pgc9s8qqaq=dirpe0xb9q8qiLsFr0=vr0=vr0dc8meaabaqaciaacaGaaeqabaqabeGadaaakeaacqWGJbWydaqhaaWcbaGaemOAaOgabaGaemODayhaaaaa@30FA@) to be rather arbitrary. The full interaction matrix of a protein structure is square and symmetric and is defined as

I^=[0F(h1,h2)⋯F(h1,hn)F(h2,h1)0⋯F(h2,hn)⋮⋮⋱⋮F(hn,h1)F(hn,h2)⋯0]N×N     (2)
 MathType@MTEF@5@5@+=feaafiart1ev1aaatCvAUfKttLearuWrP9MDH5MBPbIqV92AaeXatLxBI9gBaebbnrfifHhDYfgasaacH8akY=wiFfYdH8Gipec8Eeeu0xXdbba9frFj0=OqFfea0dXdd9vqai=hGuQ8kuc9pgc9s8qqaq=dirpe0xb9q8qiLsFr0=vr0=vr0dc8meaabaqaciaacaGaaeqabaqabeGadaaakeaaieqacuWFjbqsgaqcaiabg2da9maadmaabaqbaeqabqabaaaaaeaacqWHWaamaeaacqWHgbGrcqWHOaakcqWHObaAdaWgaaWcbaGaeGymaedabeaakiabhYcaSiabhIgaOnaaBaaaleaacqaIYaGmaeqaaOGaeCykaKcabaGaeS47IWeabaGaeCOrayKaeCikaGIaeCiAaG2aaSbaaSqaaiabigdaXaqabaGccqWHSaalcqWHObaAdaWgaaWcbaGaemOBa4gabeaakiabhMcaPaqaaiabhAeagjabhIcaOiabhIgaOnaaBaaaleaacqaIYaGmaeqaaOGaeCilaWIaeCiAaG2aaSbaaSqaaiabigdaXaqabaGccqWHPaqkaeaacqWHWaamaeaacqWIVlctaeaacqWHgbGrcqWHOaakcqWHObaAdaWgaaWcbaGaeGOmaidabeaakiabhYcaSiabhIgaOnaaBaaaleaacqWGUbGBaeqaaOGaeCykaKcabaGaeSO7I0eabaGaeSO7I0eabaGaeSy8I8eabaGaeSO7I0eabaGaeCOrayKaeCikaGIaeCiAaG2aaSbaaSqaaiabd6gaUbqabaGccqWHSaalcqWHObaAdaWgaaWcbaGaeGymaedabeaakiabhMcaPaqaaiabhAeagjabhIcaOiabhIgaOnaaBaaaleaacqWGUbGBaeqaaOGaeCilaWIaeCiAaG2aaSbaaSqaaiabikdaYaqabaGccqWHPaqkaeaacqWIVlctaeaacqWHWaamaaaacaGLBbGaayzxaaWaaSbaaSqaaiabd6eaojabgEna0kabd6eaobqabaGccaWLjaGaaCzcamaabmaabaGaeGOmaidacaGLOaGaayzkaaaaaa@7FF5@

The principle components of the interaction matrix is then obtained by orthogonal decomposition as shown below:

I^=ET[λ1λ2⋱λN]E     (3)
 MathType@MTEF@5@5@+=feaafiart1ev1aaatCvAUfKttLearuWrP9MDH5MBPbIqV92AaeXatLxBI9gBaebbnrfifHhDYfgasaacH8akY=wiFfYdH8Gipec8Eeeu0xXdbba9frFj0=OqFfea0dXdd9vqai=hGuQ8kuc9pgc9s8qqaq=dirpe0xb9q8qiLsFr0=vr0=vr0dc8meaabaqaciaacaGaaeqabaqabeGadaaakeaaieqacuWFjbqsgaqcaiabg2da9iabhweafnaaCaaaleqabaGaemivaqfaaOWaamWaaeaafaqabeabeaaaaaqaaiabeU7aSnaaBaaaleaacqaIXaqmaeqaaaGcbaaabaaabaaabaaabaGaeq4UdW2aaSbaaSqaaiabikdaYaqabaaakeaaaeaaaeaaaeaaaeaacqWIXlYtaeaaaeaaaeaaaeaaaeaacqaH7oaBdaWgaaWcbaGaemOta4eabeaaaaaakiaawUfacaGLDbaacqWHfbqrcaWLjaGaaCzcamaabmaabaGaeG4mamdacaGLOaGaayzkaaaaaa@4304@

where λ_1 _≥ λ_2 _≥ ⋯ ≥ λ_*N *_are the sorted eigenvalues, the corresponding eigenvectors are **e**_1_, **e**_2_,..., **e**_*N*_, and **E **= [**e**_1_, **e**_2_,..., **e**_*N*_] is an invertible matrix. Generally, the maximum eigenvalue, λ_1_, and its corresponding eigenvector in *N*-dimensional space encode the most dominant features in the structure and therefore can be effectively used to directly compare structures, as well as to identify the less obvious topological features common to the proteins. Since the eigenvalues depend largely on the dimension of interaction matrix, they are divided by the matrix size *N*, a treatment similar to the scaling of writhing numbers in the SGM method (Rogen P. and Fain B., 2003). In a relatively crude analysis, λ_1 _can be directly compared to infer structural similarity. This method is referred here as the Scaled Maximum Eigenvalue Comparison (SMEC).

In addition to the maximum eigenvalues, their corresponding eigenvectors can also be used to correlate similar structures. Particularly for pair-wise structure comparison, degree of similarity can be more accurately measured by comparing both eigenvalue and eigenvector. Since proteins are generally not of the same length, their eigenvectors cannot be directly correlated due to different dimensionality. Therefore, a "sliding window" approach is employed to correlate the smaller protein to all matching segments (length-wise) in the larger protein. Let us consider two proteins, A and B, having *N *and *M *secondary structure residues, respectively, and *N *≤ *M*. For the protein having shorter secondary segments, λ^A ^and e^A ^are respectively the maximum eigenvalue and its corresponding *N*-dimensional eigenvector. For the protein with more secondary structure residues, *M*-*N*+1 interaction matrices are decomposed, where (λ^B^_1_, e^B^_1_) represent the principle components of the interaction matrix constructed from secondary structure residues 1 ... *N*, (*λ*^B^_2_, e^B^_2_) are from secondary structure residues 2 ... N+1, and so on. To quantify structural similarity, we define a difference metric, *R*, between **Î **of protein A and **Î **of the *j*th matching segment of protein B as

Rj=||eA−ejB|||λA−λjB| , 1≤j≤M−N+1.     (4)
 MathType@MTEF@5@5@+=feaafiart1ev1aaatCvAUfKttLearuWrP9MDH5MBPbIqV92AaeXatLxBI9gBaebbnrfifHhDYfgasaacH8akY=wiFfYdH8Gipec8Eeeu0xXdbba9frFj0=OqFfea0dXdd9vqai=hGuQ8kuc9pgc9s8qqaq=dirpe0xb9q8qiLsFr0=vr0=vr0dc8meaabaqaciaacaGaaeqabaqabeGadaaakeaacqWGsbGudaWgaaWcbaGaemOAaOgabeaakiabg2da9iabcYha8jabcYha8jabhwgaLnaaCaaaleqabaGaemyqaeeaaOGaeyOeI0IaeCyzau2aa0baaSqaaiabdQgaQbqaaiabdkeacbaakiabcYha8jabcYha8naaemaabaGaeq4UdW2aaWbaaSqabeaacqWGbbqqaaGccqGHsislcqaH7oaBdaqhaaWcbaGaemOAaOgabaGaemOqaieaaaGccaGLhWUaayjcSdGaeeiiaaIaeeilaWIaeeiiaaIaeGymaeJaeyizImQaemOAaOMaeyizImQaemyta0KaeyOeI0IaemOta4Kaey4kaSIaeGymaeJaeiOla4IaaCzcaiaaxMaadaqadaqaaiabisda0aGaayjkaiaawMcaaaaa@5B1C@

Obviously, smaller *R*_*j *_indicates better correlation or higher degree of structural similarity. The overall difference between the two proteins is defined as

*R *= min(*R*_1_, *R*_2_,..., *R*_*M*-*N*+1_).     (5)

The minimum of *R*_1_, *R*_2_, ..., *R*_*M*-*N*+1 _is used here to measure similarity because this potentially allows mapping a smaller structure onto a homologous domain within a larger protein. This method is called the Principle Component Correlation (PCC) analysis.

### Defining the matrix elements

**Figure 1:**
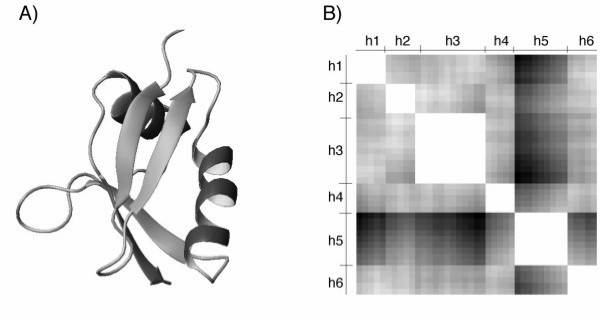
(a) Ribbon representation of 1IP9, showing two α helixes and four β strands, and (b) the corresponding symmetric interaction matrix (defined in eq. 2), where **h**_3 _and **h**_5 _are the two α helices, and **h**_1_, **h**_2_, **h**_4 _and **h**_6 _are the four β strands. The gray-level values denote the distance between any two C^α ^atoms with white corresponding to the shortest distance, i.e., 0.

The definition of block matrix elements, *d*(ciu
 MathType@MTEF@5@5@+=feaafiart1ev1aaatCvAUfKttLearuWrP9MDH5MBPbIqV92AaeXatLxBI9gBaebbnrfifHhDYfgasaacH8akY=wiFfYdH8Gipec8Eeeu0xXdbba9frFj0=OqFfea0dXdd9vqai=hGuQ8kuc9pgc9s8qqaq=dirpe0xb9q8qiLsFr0=vr0=vr0dc8meaabaqaciaacaGaaeqabaqabeGadaaakeaacqWGJbWydaqhaaWcbaGaemyAaKgabaGaemyDauhaaaaa@30F6@, cjv
 MathType@MTEF@5@5@+=feaafiart1ev1aaatCvAUfKttLearuWrP9MDH5MBPbIqV92AaeXatLxBI9gBaebbnrfifHhDYfgasaacH8akY=wiFfYdH8Gipec8Eeeu0xXdbba9frFj0=OqFfea0dXdd9vqai=hGuQ8kuc9pgc9s8qqaq=dirpe0xb9q8qiLsFr0=vr0=vr0dc8meaabaqaciaacaGaaeqabaqabeGadaaakeaacqWGJbWydaqhaaWcbaGaemOAaOgabaGaemODayhaaaaa@30FA@), depends on the desired structural features to be extracted. In the current study, we focus structural comparison on protein backbone conformation. Clearly the simplest invariant describing the backbone conformation is the Euclidian distance between a pair of C^α ^atoms from two different secondary segments. Formally, the elements are defined as *d*(ciu
 MathType@MTEF@5@5@+=feaafiart1ev1aaatCvAUfKttLearuWrP9MDH5MBPbIqV92AaeXatLxBI9gBaebbnrfifHhDYfgasaacH8akY=wiFfYdH8Gipec8Eeeu0xXdbba9frFj0=OqFfea0dXdd9vqai=hGuQ8kuc9pgc9s8qqaq=dirpe0xb9q8qiLsFr0=vr0=vr0dc8meaabaqaciaacaGaaeqabaqabeGadaaakeaacqWGJbWydaqhaaWcbaGaemyAaKgabaGaemyDauhaaaaa@30F6@, cjv
 MathType@MTEF@5@5@+=feaafiart1ev1aaatCvAUfKttLearuWrP9MDH5MBPbIqV92AaeXatLxBI9gBaebbnrfifHhDYfgasaacH8akY=wiFfYdH8Gipec8Eeeu0xXdbba9frFj0=OqFfea0dXdd9vqai=hGuQ8kuc9pgc9s8qqaq=dirpe0xb9q8qiLsFr0=vr0=vr0dc8meaabaqaciaacaGaaeqabaqabeGadaaakeaacqWGJbWydaqhaaWcbaGaemOAaOgabaGaemODayhaaaaa@30FA@) = ||ciu
 MathType@MTEF@5@5@+=feaafiart1ev1aaatCvAUfKttLearuWrP9MDH5MBPbIqV92AaeXatLxBI9gBaebbnrfifHhDYfgasaacH8akY=wiFfYdH8Gipec8Eeeu0xXdbba9frFj0=OqFfea0dXdd9vqai=hGuQ8kuc9pgc9s8qqaq=dirpe0xb9q8qiLsFr0=vr0=vr0dc8meaabaqaciaacaGaaeqabaqabeGadaaakeaacqWGJbWydaqhaaWcbaGaemyAaKgabaGaemyDauhaaaaa@30F6@ - cjv
 MathType@MTEF@5@5@+=feaafiart1ev1aaatCvAUfKttLearuWrP9MDH5MBPbIqV92AaeXatLxBI9gBaebbnrfifHhDYfgasaacH8akY=wiFfYdH8Gipec8Eeeu0xXdbba9frFj0=OqFfea0dXdd9vqai=hGuQ8kuc9pgc9s8qqaq=dirpe0xb9q8qiLsFr0=vr0=vr0dc8meaabaqaciaacaGaaeqabaqabeGadaaakeaacqWGJbWydaqhaaWcbaGaemOAaOgabaGaemODayhaaaaa@30FA@|| where ciu
 MathType@MTEF@5@5@+=feaafiart1ev1aaatCvAUfKttLearuWrP9MDH5MBPbIqV92AaeXatLxBI9gBaebbnrfifHhDYfgasaacH8akY=wiFfYdH8Gipec8Eeeu0xXdbba9frFj0=OqFfea0dXdd9vqai=hGuQ8kuc9pgc9s8qqaq=dirpe0xb9q8qiLsFr0=vr0=vr0dc8meaabaqaciaacaGaaeqabaqabeGadaaakeaacqWGJbWydaqhaaWcbaGaemyAaKgabaGaemyDauhaaaaa@30F6@ and cjv
 MathType@MTEF@5@5@+=feaafiart1ev1aaatCvAUfKttLearuWrP9MDH5MBPbIqV92AaeXatLxBI9gBaebbnrfifHhDYfgasaacH8akY=wiFfYdH8Gipec8Eeeu0xXdbba9frFj0=OqFfea0dXdd9vqai=hGuQ8kuc9pgc9s8qqaq=dirpe0xb9q8qiLsFr0=vr0=vr0dc8meaabaqaciaacaGaaeqabaqabeGadaaakeaacqWGJbWydaqhaaWcbaGaemOAaOgabaGaemODayhaaaaa@30FA@ are the coordinates of the two C^α ^atoms of residues *u *of **h**_i _and *v *of **h**_j_, respectively. For conciseness, we name the interaction matrix so defined as the Pair-wise Distance (PD) matrix. For illustration purpose, the interaction matrix for the structure of Pb1, Domain of Bem1P (PDB accession code 1IP9), is shown in Fig. 1. This structure, consisting of two α helices and four β strands (Fig. 1a), is used here to provide distances between all pairs of C_α _atoms in the six secondary elements (Fig. 1b).

Furthermore, two variations of the PD matrix definition are explored in attempt to provide a better resolution in structural comparison and classification. Since physical energy of interaction between a pair of atoms typically increase monotonically as the inverse of their separation, inverse of distance is used to mimic physical interactions between secondary elements. Here the elements of **F**(**h**_*i*_, **h**_*j*_) are defined as

d(ciu,cjv)={1||ciu−cjv||,||ciu−cjv||≥u01u0||ciu−cjv||<u0     (6)
 MathType@MTEF@5@5@+=feaafiart1ev1aaatCvAUfKttLearuWrP9MDH5MBPbIqV92AaeXatLxBI9gBaebbnrfifHhDYfgasaacH8akY=wiFfYdH8Gipec8Eeeu0xXdbba9frFj0=OqFfea0dXdd9vqai=hGuQ8kuc9pgc9s8qqaq=dirpe0xb9q8qiLsFr0=vr0=vr0dc8meaabaqaciaacaGaaeqabaqabeGadaaakeaacqWGKbazcqGGOaakcqWGJbWydaqhaaWcbaGaemyAaKgabaGaemyDauhaaOGaeiilaWIaem4yam2aa0baaSqaaiabdQgaQbqaaiabdAha2baakiabcMcaPiabg2da9maaceaabaqbaeqabiGaaaqaamaalaaabaGaeGymaedabaWaaqWaaeaadaabdaqaaiabdogaJnaaDaaaleaacqWGPbqAaeaacqWG1bqDaaGccqGHsislcqWGJbWydaqhaaWcbaGaemOAaOgabaGaemODayhaaaGccaGLhWUaayjcSdaacaGLhWUaayjcSdaaaiabcYcaSaqaamaaemaabaWaaqWaaeaacqWGJbWydaqhaaWcbaGaemyAaKgabaGaemyDauhaaOGaeyOeI0Iaem4yam2aa0baaSqaaiabdQgaQbqaaiabdAha2baaaOGaay5bSlaawIa7aaGaay5bSlaawIa7aiabgwMiZkabdwha1naaBaaaleaacqaIWaamaeqaaaGcbaWaaSaaaeaacqaIXaqmaeaacqWG1bqDdaWgaaWcbaGaeGimaadabeaaaaaakeaadaabdaqaamaaemaabaGaem4yam2aa0baaSqaaiabdMgaPbqaaiabdwha1baakiabgkHiTiabdogaJnaaDaaaleaacqWGQbGAaeaacqWG2bGDaaaakiaawEa7caGLiWoaaiaawEa7caGLiWoacqGH8aapcqWG1bqDdaWgaaWcbaGaeGimaadabeaaaaaakiaawUhaaiaaxMaacaWLjaWaaeWaaeaacqaI2aGnaiaawIcacaGLPaaaaaa@7C38@

where *u*_0 _represent a hard-sphere boundary below which the interaction is constant. In this study, we arbitrarily set *u*_0 _to 3**Å**. This definition is referred as Pair-wise Inverse Distance (PID) matrix.

Another variation of the PD matrix definition is to take into account the N – C terminal sense, in attempt to further emphasize protein topological features. For a secondary element, **h**_*i*_, its direction vector **v**_*i *_is defined by two points in Cartesian space: the center of mass of the five consecutive N-terminal C^α ^and the center of mass of the five consecutive C-terminal C^α ^atoms. Given a pair of secondary elements **h**_*i *_and **h**_*j*_, the new matrix elements are defined as

*d*(ciu
 MathType@MTEF@5@5@+=feaafiart1ev1aaatCvAUfKttLearuWrP9MDH5MBPbIqV92AaeXatLxBI9gBaebbnrfifHhDYfgasaacH8akY=wiFfYdH8Gipec8Eeeu0xXdbba9frFj0=OqFfea0dXdd9vqai=hGuQ8kuc9pgc9s8qqaq=dirpe0xb9q8qiLsFr0=vr0=vr0dc8meaabaqaciaacaGaaeqabaqabeGadaaakeaacqWGJbWydaqhaaWcbaGaemyAaKgabaGaemyDauhaaaaa@30F6@, cjv
 MathType@MTEF@5@5@+=feaafiart1ev1aaatCvAUfKttLearuWrP9MDH5MBPbIqV92AaeXatLxBI9gBaebbnrfifHhDYfgasaacH8akY=wiFfYdH8Gipec8Eeeu0xXdbba9frFj0=OqFfea0dXdd9vqai=hGuQ8kuc9pgc9s8qqaq=dirpe0xb9q8qiLsFr0=vr0=vr0dc8meaabaqaciaacaGaaeqabaqabeGadaaakeaacqWGJbWydaqhaaWcbaGaemOAaOgabaGaemODayhaaaaa@30FA@)' = *d*(ciu
 MathType@MTEF@5@5@+=feaafiart1ev1aaatCvAUfKttLearuWrP9MDH5MBPbIqV92AaeXatLxBI9gBaebbnrfifHhDYfgasaacH8akY=wiFfYdH8Gipec8Eeeu0xXdbba9frFj0=OqFfea0dXdd9vqai=hGuQ8kuc9pgc9s8qqaq=dirpe0xb9q8qiLsFr0=vr0=vr0dc8meaabaqaciaacaGaaeqabaqabeGadaaakeaacqWGJbWydaqhaaWcbaGaemyAaKgabaGaemyDauhaaaaa@30F6@, cjv
 MathType@MTEF@5@5@+=feaafiart1ev1aaatCvAUfKttLearuWrP9MDH5MBPbIqV92AaeXatLxBI9gBaebbnrfifHhDYfgasaacH8akY=wiFfYdH8Gipec8Eeeu0xXdbba9frFj0=OqFfea0dXdd9vqai=hGuQ8kuc9pgc9s8qqaq=dirpe0xb9q8qiLsFr0=vr0=vr0dc8meaabaqaciaacaGaaeqabaqabeGadaaakeaacqWGJbWydaqhaaWcbaGaemOAaOgabaGaemODayhaaaaa@30FA@)sgn(**v**_*i*_·**v**_*j*_)     (7)

where sgn(*x*) is a symbol function which is 1 when *x *≥ 0 and -1 when *x *< 0. This variation is referred as Pair-wise Distance with Sense (PDS) matrix in this study.

### Linking/Writhing numbers

To evaluate the ability of PCC analysis in extracting pure topological features, the linking and writhing numbers, which are good measures of global topology, are also calculated for the four sets of structures for comparison. The linking number of two curves is defined by the C**ă**lug**ă**reanu-Fuller-White formula [25-27]: *Lk *= *Wr *+ *Tw*, where the linking number *Lk *counts the sum of signed crossings between the ribbon's two boundary curves, the writhing number *Wr *counts the sum of signed self-crossings of the curve, averaged over all projection directions [28], and *Tw *is the twist number.*Lk *is an invariant to any smooth deformation that avoids self-intersections [29], and it is also independent of projection direction. *Wr *and *Tw *are invariant to some transformations, such as rigid body motions. Here we compute the writhing numbers using the Scaled Gauss Metric (SGM) approach previously described by Rogen and Fain [22].

Given two curves *c*_1 _and *c*_2_, which are two closed non-intersecting curves in 3-dimentional space, and define *e*(*s*, *t*) = (*c*_2_(*t*) - *c*_1_(*s*))/||*c*_2_(*t*) - *c*_1_(*s*)||, where ||·|| denotes the Euclidean norm. For two closed curves, the vector field *e*(*s*, *t*) is doubly periodic. Such mappings have an integer-valued degree that is invariant under topological deformations. The linking number of two curves is further defined as

Lk(c1,c)2=14π∫c1∫c2[e,es,et]dsdt=14π∫c1∫c2(c1'(s)×c2'(t))⋅(c1(s)−c2(t))‖c1(s)−c2(t)‖3dsdt     (8)
 MathType@MTEF@5@5@+=feaafiart1ev1aaatCvAUfKttLearuWrP9MDH5MBPbIqV92AaeXatLxBI9gBaebbnrfifHhDYfgasaacH8akY=wiFfYdH8Gipec8Eeeu0xXdbba9frFj0=OqFfea0dXdd9vqai=hGuQ8kuc9pgc9s8qqaq=dirpe0xb9q8qiLsFr0=vr0=vr0dc8meaabaqaciaacaGaaeqabaqabeGadaaakeaacqWGmbatcqWGRbWAcqGGOaakcqWGJbWydaWgaaWcbaGaeGymaedabeaakiabcYcaSiabdogaJnaaBeaaleaacqaIYaGmaeqaaOGaeiykaKIaeyypa0ZaaSaaaeaacqaIXaqmaeaacqaI0aancqaHapaCaaWaa8qeaeaadaWdraqaamaadmaabaGaemyzauMaeiilaWIaemyzau2aaSbaaSqaaiabdohaZbqabaGccqGGSaalcqWGLbqzdaWgaaWcbaGaemiDaqhabeaaaOGaay5waiaaw2faaaWcbaGaem4yam2aaSbaaWqaaiabikdaYaqabaaaleqaniabgUIiYdaaleaacqWGJbWydaWgaaadbaGaeGymaedabeaaaSqab0Gaey4kIipakiabdsgaKjabdohaZjabdsgaKjabdsha0jabg2da9maalaaabaGaeGymaedabaGaeGinaqJaeqiWdahaamaapebabaWaa8qeaeaadaWcaaqaamaabmaabaGaem4yam2aa0baaSqaaiabigdaXaqaaiabcEcaNaaakiabcIcaOiabdohaZjabcMcaPiabgEna0kabdogaJnaaDaaaleaacqaIYaGmaeaacqGGNaWjaaGccqGGOaakcqWG0baDcqGGPaqkaiaawIcacaGLPaaacqGHflY1daqadaqaaiabdogaJnaaBaaaleaacqaIXaqmaeqaaOGaeiikaGIaem4CamNaeiykaKIaeyOeI0Iaem4yam2aaSbaaSqaaiabikdaYaqabaGccqGGOaakcqWG0baDcqGGPaqkaiaawIcacaGLPaaaaeaadaqbdaqaaiabdogaJnaaBaaaleaacqaIXaqmaeqaaOGaeiikaGIaem4CamNaeiykaKIaeyOeI0Iaem4yam2aaSbaaSqaaiabikdaYaqabaGccqGGOaakcqWG0baDcqGGPaqkaiaawMa7caGLkWoadaahaaWcbeqaaiabiodaZaaaaaaabaGaem4yam2aaSbaaWqaaiabikdaYaqabaaaleqaniabgUIiYdaaleaacqWGJbWydaWgaaadbaGaeGymaedabeaaaSqab0Gaey4kIipakiabdsgaKjabdohaZjabdsgaKjabdsha0jaaxMaacaWLjaWaaeWaaeaacqaI4aaoaiaawIcacaGLPaaaaaa@9CD7@

where *e*_*s *_and *e*_*t *_are the tangents of *e*(*s*, *t*) at point (*s*, *t*), as well as c1'
 MathType@MTEF@5@5@+=feaafiart1ev1aaatCvAUfKttLearuWrP9MDH5MBPbIqV92AaeXatLxBI9gBaebbnrfifHhDYfgasaacH8akY=wiFfYdH8Gipec8Eeeu0xXdbba9frFj0=OqFfea0dXdd9vqai=hGuQ8kuc9pgc9s8qqaq=dirpe0xb9q8qiLsFr0=vr0=vr0dc8meaabaqaciaacaGaaeqabaqabeGadaaakeaacqWGJbWydaqhaaWcbaGaeGymaedabaGaei4jaCcaaaaa@2FEE@(*s*) and c2'
 MathType@MTEF@5@5@+=feaafiart1ev1aaatCvAUfKttLearuWrP9MDH5MBPbIqV92AaeXatLxBI9gBaebbnrfifHhDYfgasaacH8akY=wiFfYdH8Gipec8Eeeu0xXdbba9frFj0=OqFfea0dXdd9vqai=hGuQ8kuc9pgc9s8qqaq=dirpe0xb9q8qiLsFr0=vr0=vr0dc8meaabaqaciaacaGaaeqabaqabeGadaaakeaacqWGJbWydaqhaaWcbaGaeGOmaidabaGaei4jaCcaaaaa@2FF0@(*t*) are the tangents along the *c*_1 _and *c*_2 _at *s *and *t*. Note that here *e*_*s*_, *e*_*t*_, c1'
 MathType@MTEF@5@5@+=feaafiart1ev1aaatCvAUfKttLearuWrP9MDH5MBPbIqV92AaeXatLxBI9gBaebbnrfifHhDYfgasaacH8akY=wiFfYdH8Gipec8Eeeu0xXdbba9frFj0=OqFfea0dXdd9vqai=hGuQ8kuc9pgc9s8qqaq=dirpe0xb9q8qiLsFr0=vr0=vr0dc8meaabaqaciaacaGaaeqabaqabeGadaaakeaacqWGJbWydaqhaaWcbaGaeGymaedabaGaei4jaCcaaaaa@2FEE@(*s*), and c2'
 MathType@MTEF@5@5@+=feaafiart1ev1aaatCvAUfKttLearuWrP9MDH5MBPbIqV92AaeXatLxBI9gBaebbnrfifHhDYfgasaacH8akY=wiFfYdH8Gipec8Eeeu0xXdbba9frFj0=OqFfea0dXdd9vqai=hGuQ8kuc9pgc9s8qqaq=dirpe0xb9q8qiLsFr0=vr0=vr0dc8meaabaqaciaacaGaaeqabaqabeGadaaakeaacqWGJbWydaqhaaWcbaGaeGOmaidabaGaei4jaCcaaaaa@2FF0@(*t*) are vectors. Define *w*(*s*, *t*) = (*c*_1_(*t*) - *c*_1_(*s*)/||*c*_1_(*t*) - *c*_1_(*s*)||. The writhing number for a single curve *c*_1 _is defined as

Wr(c1)=14π∫c1∫c1[w,ws,wt]dsdt=14π∫c1∫c2(c1'(s)×c1'(t))⋅(c1(s)−c1(t))‖c1(s)−c1(t)‖3dsdt     (9)
 MathType@MTEF@5@5@+=feaafiart1ev1aaatCvAUfKttLearuWrP9MDH5MBPbIqV92AaeXatLxBI9gBaebbnrfifHhDYfgasaacH8akY=wiFfYdH8Gipec8Eeeu0xXdbba9frFj0=OqFfea0dXdd9vqai=hGuQ8kuc9pgc9s8qqaq=dirpe0xb9q8qiLsFr0=vr0=vr0dc8meaabaqaciaacaGaaeqabaqabeGadaaakeaacqWGxbWvcqWGYbGCcqGGOaakcqWGJbWydaWgaaWcbaGaeGymaedabeaakiabcMcaPiabg2da9maalaaabaGaeGymaedabaGaeGinaqJaeqiWdahaamaapebabaWaa8qeaeaadaWadaqaaiabdEha3jabcYcaSiabdEha3naaBaaaleaacqWGZbWCaeqaaOGaeiilaWIaem4DaC3aaSbaaSqaaiabdsha0bqabaaakiaawUfacaGLDbaaaSqaaiabdogaJnaaBaaameaacqaIXaqmaeqaaaWcbeqdcqGHRiI8aaWcbaGaem4yam2aaSbaaWqaaiabigdaXaqabaaaleqaniabgUIiYdGccqWGKbazcqWGZbWCcqWGKbazcqWG0baDcqGH9aqpdaWcaaqaaiabigdaXaqaaiabisda0iabec8aWbaadaWdraqaamaapebabaWaaSaaaeaadaqadaqaaiabdogaJnaaDaaaleaacqaIXaqmaeaacqGGNaWjaaGccqGGOaakcqWGZbWCcqGGPaqkcqGHxdaTcqWGJbWydaqhaaWcbaGaeGymaedabaGaei4jaCcaaOGaeiikaGIaemiDaqNaeiykaKcacaGLOaGaayzkaaGaeyyXIC9aaeWaaeaacqWGJbWydaWgaaWcbaGaeGymaedabeaakiabcIcaOiabdohaZjabcMcaPiabgkHiTiabdogaJnaaBaaaleaacqaIXaqmaeqaaOGaeiikaGIaemiDaqNaeiykaKcacaGLOaGaayzkaaaabaWaauWaaeaacqWGJbWydaWgaaWcbaGaeGymaedabeaakiabcIcaOiabdohaZjabcMcaPiabgkHiTiabdogaJnaaBaaaleaacqaIXaqmaeqaaOGaeiikaGIaemiDaqNaeiykaKcacaGLjWUaayPcSdWaaWbaaSqabeaacqaIZaWmaaaaaaqaaiabdogaJnaaBaaameaacqaIYaGmaeqaaaWcbeqdcqGHRiI8aaWcbaGaem4yam2aaSbaaWqaaiabigdaXaqabaaaleqaniabgUIiYdGccqWGKbazcqWGZbWCcqWGKbazcqWG0baDcaWLjaGaaCzcamaabmaabaGaeGyoaKdacaGLOaGaayzkaaaaaa@9A09@

where *w*_*s *_and *w*_*t *_are the tangent of *w*(*s*, *t*) at point (*s*, *t*). Writhing number is not invariant under general smooth deformations such as translations, rotations, re-parameterizations, and dilations (Murasugi, 1996). Since the backbone of a protein is a polygonal curve, the writhing number of *c*_1_(*t*) can be calculated by

Wr(c1)=∑0<i1<i2<NW(i1,i2),         W(i1,i2)=12π∫i1=ss+1∫i2=tt+1w(s,t)dsdt     (10)
 MathType@MTEF@5@5@+=feaafiart1ev1aaatCvAUfKttLearuWrP9MDH5MBPbIqV92AaeXatLxBI9gBaebbnrfifHhDYfgasaacH8akY=wiFfYdH8Gipec8Eeeu0xXdbba9frFj0=OqFfea0dXdd9vqai=hGuQ8kuc9pgc9s8qqaq=dirpe0xb9q8qiLsFr0=vr0=vr0dc8meaabaqaciaacaGaaeqabaqabeGadaaakeaacqWGxbWvcqWGYbGCcqGGOaakcqWGJbWydaWgaaWcbaGaeGymaedabeaakiabcMcaPiabg2da9maaqafabaGaem4vaCLaeiikaGIaemyAaK2aaSbaaSqaaiabigdaXaqabaGccqGGSaalcqWGPbqAdaWgaaWcbaGaeGOmaidabeaakiabcMcaPiabcYcaSiabbccaGiabbccaGiabbccaGiabbccaGiabbccaGiabbccaGiabbccaGiabbccaGiabbccaGiabdEfaxjabcIcaOiabdMgaPnaaBaaaleaacqaIXaqmaeqaaOGaeiilaWIaemyAaK2aaSbaaSqaaiabikdaYaqabaGccqGGPaqkcqGH9aqpdaWcaaqaaiabigdaXaqaaiabikdaYiabec8aWbaadaWdXaqaamaapedabaGaem4DaCNaeiikaGIaem4CamNaeiilaWIaemiDaqNaeiykaKIaemizaqMaem4CamNaemizaqMaemiDaqhaleaacqWGPbqAdaWgaaadbaGaeGOmaidabeaaliabg2da9iabdsha0bqaaiabdsha0jabgUcaRiabigdaXaqdcqGHRiI8aaWcbaGaemyAaK2aaSbaaWqaaiabigdaXaqabaWccqGH9aqpcqWGZbWCaeaacqWGZbWCcqGHRaWkcqaIXaqma0Gaey4kIipaaSqaaiabicdaWiabgYda8iabdMgaPnaaBaaameaacqaIXaqmaeqaaSGaeyipaWJaemyAaK2aaSbaaWqaaiabikdaYaqabaWccqGH8aapcqWGobGtaeqaniabggHiLdGccaWLjaGaaCzcamaabmaabaGaeGymaeJaeGimaadacaGLOaGaayzkaaaaaa@84DF@

where *W*(*i*_1_, *i*_2_) is the writhing number between the *i*_1 _th and the *i*_2_th segment; *s *and *t *denote two different C^α ^atoms, and *N *is the total number of C^α ^atoms. The SGM method is defined as the normalized writhing number, namely, *Wr *is divided by *N *[22]. The absolute difference between their writhing numbers is used to infer topological similarity.

## Authors' contributions

X.Z and J.C. played the major role in carrying out the proposed approach and experiments, as well as drafted the manuscript. S.T.C.W. has been involved in and has guided the research discussion, as well as the preparation of the manuscript. He has given the final approval of the version to be published.
